# Menopause, skin and common dermatoses. Part 4: oral disorders

**DOI:** 10.1111/ced.15341

**Published:** 2022-11-13

**Authors:** Mariha Ashraf, Erin Kamp, Esra Musbahi, Claudia DeGiovanni

**Affiliations:** ^1^ Department of Dermatology University Hospitals Sussex NHS Foundation Trust Brighton UK

## Abstract

The physiological impact of declining oestrogen levels during menopause has been well documented. We conducted a literature review to assess the impact of menopause on oral health. Falling oestrogen levels are associated with adverse effects on the gingival, oral and buccal epithelia. The symptoms prevalent in perimenopausal and postmenopausal women range from dry mouth to immune‐mediated mucocutaneous disease and burning mouth syndrome. Our review has highlighted the need for further research into potential treatments for oral symptoms in menopause, particularly with regard to hormone replacement therapy.

## Introduction

Menopause is defined as a point 12 months after a woman's final period, and starts on average between the ages of 45 and 55 years.^1^ Declining oestrogen levels lead to several physiological changes in a woman's body, including changes to the oral cavity. In this review, we aim to explore the variety of oral symptoms that occur during the menopause, and to highlight areas requiring further research and exploration.

## Search strategy

The Cochrane Library, National Institute for Health and Care Excellence (NICE) Evidence database and the Turning Research into Practice database were searched from 2001 to 2021. In total, 116 original research articles were found on menopause in dermatology, 13 of which related to oral health in menopause. Individual searches were performed for specific queries related to our paper.

## Oestrogen and the mouth

Oestrogen is well known for its role in cell growth and regulation, and acts through oestrogen receptors (ERs), which are found throughout the body. There are two different subtypes of ERs: ER‐α is found in oestrogen target tissues and in the mouth, while ER‐β is the predominant receptor found in the gingiva, buccal epithelia and salivary glands.^2^ Fluctuations in hormone levels have been associated with pathological processes affecting regions with high expression of these receptors. This results in thinning of the buccal epithelium, reduction in salivary flow and reduction in bone density^3^ (Table 1).

**Table 1:** Effects of sex steroids in the mouth^3^, ^26^ and on immune system regulation.^26^, ^27^, ^28^, ^29^

Hormone	Effects	
	Mouth	Immune system regulation
Oestrogen	Regulates bone turnover through osteoblasts, osteoclasts and osteocytes Antiresorptive effect on periodontal tissues Synthesis and maintenance of fibrous collagen Stimulates synthesis of gingival fibroblasts	Increases anti‐inflammatory cytokines (IL‐10, TGF‐β) Reduces proinflammatory cytokines (IL‐1, IL‐6, TNF‐α) Reduces B‐cell apoptosis and alters B‐cell maturation Promotes dendritic cell maturation Has direct antioxidant effects
Progesterone	Increases vascular permeability and dilatation in periodontal tissue Inhibits proliferation of gingival fibroblasts Stimulates production of the inflammatory mediator prostaglandin E_2_	Involved in downregulation of proinflammatory cytokines from gingival fibroblasts Anti‐inflammatory properties, promoting Treg differentiation and anti‐inflammatory cytokine production Regulates T‐cell homeostasis through inducing T‐cell apoptosis
Androgens	Regulates bone turnover through increased osteoblast synthesis Reduces inflammatory prostaglandin production	Immunosuppressive effects through downregulation of natural killer cell response Increases activity of Th1 and anti‐inflammatory cytokines (IL‐10) Downregulates proinflammatory cytokines during inflammation (IL‐6) Suppresses B lymphopoiesis

IL, interleukin; TGF, transforming growth factor; Th, T helper; TNF, tumour necrosis factor; Treg, regulatory T cell.

## Oral epithelia

Oestrogen plays a role in angiogenesis, vascular permeability and fibroblast mediation. Fluctuations in oestrogen levels and bone mineral density have been found to exaggerate the loss of alveolar bone, causing deterioration of periodontal health and ultimately leading to tooth loss.^4^ Passos‐Soares *et al*.^5^ demonstrated that postmenopausal women treated with oestrogen replacement for postmenopausal osteoporosis subsequently had a lower prevalence of severe periodontitis than a control group of women who received no treatment.

A systematic review by Allen *et al*.^6^ assessed the health and economic outcomes of hormone replacement therapy (HRT) in dental care for postmenopausal women, and found consistently improved dental outcomes in the HRT group compared with the non‐HRT group. However, most of the studies included in this review were single‐arm trials or observational designs, and there was a lack of large, randomized studies.

Microscopic examination of the buccal and vaginal epithelia has demonstrated structural similarities.^7^ Although changes in the vaginal microbiome during menopause have been observed, the oral microbiome has not been examined as extensively. Given the histological similarities between the oral and vaginal epithelium, further research is required to establish if there are any changes in the oral microbiome with menopause. An observational case–control trial is currently being undertaken, evaluating dental plaque and the saliva microbiome in premenopausal and postmenopausal women.^8^

## Salivary glands

### Salivary flow

Saliva is necessary for maintaining oral health, facilitating digestion and providing an antimicrobial defence mechanism.^9^ The salivary glands contain ERs and therefore could be vulnerable to changes in circulating oestrogen levels. A number of studies have found postmenopausal women to have lower salivary flow rates compared with menstruating women,^10^, ^11^ predisposing to dental plaque, oral infections (such as candidiasis) and taste disturbance.^4^, ^12^ The saliva is also subject to changes in composition during menopause, including increased concentrations of salivary calcium, which carries potential implications such as faster mineralization of plaques and increased susceptibility to periodontitis.^4^

### Xerostomia

Xerostomia is the subjective feeling of dry mouth while hyposalivation is the objective reduction in salivary flow.^13^ Xerostomia and reduced saliva flow are both well‐recognized symptoms among menopausal women.^14^ Interestingly a study by Minicucci *et al*.^11^ found that although salivary flow rates were reduced in postmenopausal women, there was no association with the sensation of dry mouth. The cause of xerostomia is multifactorial, and currently no topical therapy has proven to be most effective in reducing the symptoms of dry mouth.^13^, ^15^ A recent case–control study conducted by Wang *et al*.^16^ found a reduction in dry mouth symptoms following HRT, but no improvements in burning sensations or waking up thirsty at night. However, the study had a small sample size and carried the possibility of bias through subjective questionnaire responses.

## Immune‐mediated conditions

Immune‐mediated mucocutaneous diseases with oral involvement are generally more prevalent in women in the fourth and fifth decades of life.^17^ Sex steroids play an important role in immune system development and regulation (Table 1).

### Oral lichen planus

Oral lichen planus (OLP) is an inflammatory autoimmune disease of the mucous membranes. The current literature shows that OLP is more prevalent in women and in people aged > 40 years.^18^ One study found a higher incidence of OLP in perimenopausal compared with premenopausal women (10.9% compared with 0.5–2%).^4^ Patients with the ulcerated, erosive or bullous form of lichen planus have a significantly reduced quality of life due to pain.^19^

Management of OLP includes a variety of topical, systemic and nonpharmacological preparations, listed in Table 2. A systematic review conducted by Oberti *et al*.^20^ found the most effective first‐line treatment for OLP was topical corticosteroids, while for management of refractory cases, topical calcineurin inhibitors were effective as second‐line treatment. For highly active, erosive OLP, topical isotretinoin in high concentrations as a cream, gel or ointment may be effective; however, rapid recurrence after treatment cessation has been reported.^21^, ^22^ Despite the evidence demonstrating that sex steroids reduce inflammation in the mouth (Table 3), the effect of HRT as a treatment for OLP has not been explored sufficiently.

**Table 2:** Reported treatment options for oral lichen planus.^20^, ^21^, ^22^, ^30^

Topical/localized	Systemic	Alternative
Corticosteroids (first‐line) as drops, paste or gels on average for 1 month, 2–4 times daily: triamcinolone acetonide 0.1%; clobetasol proprionate 0.05%; dexamethasone 0.05% Intralesional corticosteroid injections Calcineurin inhibitors (second‐line): tacrolimus 0.1%, pimecrolimus 1% 2–4 times daily Retinoid: isotretinoin 0.05%, 0.1% or 0.18% as cream, gel or ointment Ciclosporin: topical or rinse 2 times daily	Oral corticosteroids, prednisolone short course Mycophenolate mofetil 0.5–3 g/day Azathioprine 100–150 mg/day Dapsone 50–100 mg/day Methotrexate 2.5–12.5 mg/week Curcumin 6 g/day Biologic agents, e.g. TNF‐α inhibitors and rituximab	PDT Cryotherapy CO_2_ laser Excisional surgery Aloe vera

PDT, photodynamic therapy; TNF, tumour necrosis factor.

**Table 3:** Potential treatments for burning mouth syndrome.^31^, ^32^

Treatment
ALA 200 mg three times daily
Topical clonazepam 1 mg for 3 min three times daily
Gabapentin 300 mg/day up to 300 mg three times daily for 2 months
Combination ALA 600 mg/day and gabapentin 300 mg/day for 2 months

ALA, α‐lipoic acid.

### Burning mouth syndrome

Burning mouth syndrome (BMS) is a multifactorial illness affecting approximately 10–40% of perimenopausal women. It is characterized by a burning sensation in the anterior aspect of the tongue, hard palate or lower lip mucosa.^23^ The symptoms range from mild to severe and resolve spontaneously in around half of affected individuals.^24^ Management of BMS remains a challenge as further research is required to truly understand the efficacy of treatment. Differential diagnoses for BMS such as oral candida should be excluded.^25^ Although the published studies are small, the therapeutic options are listed in Table 3.

## Conclusion

The physiological impact of the menopause on oral health often causes chronic discomfort and a reduced quality of life (QoL). Symptoms include dry mouth, increased oral infections and deterioration of periodontal health. What has been highlighted from our review is the need for large, longer‐term randomized controlled trials (RCTs) to fully assess the management of oral symptoms and the potential role of HRT. Dermatologists should be aware of the potential implications of menopause on oral health, and encourage patients to maintain routine appointments with dentists and practise good oral hygiene.

This series on menopause has highlighted the multifaceted effect of menopause on common dermatoses and the associated adversarial effect on the QoL of menopausal women. Understanding the mechanism of fluctuating hormone levels and its cutaneous manifestation, could lead to better targeted therapy in the future. We have demonstrated a need for large, long‐term RCTs on the potential role of HRT as a therapeutic option for hair, skin, vulval and oral health.

**Learning points**

- There is a significant psychosocial impact oral discomfort can have on women during the premenopausal and postmenopausal period.
- Given the similarities between the vaginal and oral mucosa, more research is needed on the effect of the oral microbiome during menopause.
- Reduced salivary flow, xerostomia and BMS are all associated with the postmenopausal state and can have a significant impact on QoL.
- Immune‐mediated mucocutaneous diseases are more prevalent in women in their fourth and fifth decades.
- Dermatologists should ensure menopausal patients are seeking regular dental care and practising good oral hygiene.
- The use of HRT for oral discomfort seen in menopause requires further research in the form of large RCTs.

## Conflict of interest

The authors declare that they have no conflicts of interest.

## Funding

Funding for open access was provided by the University of Sussex as CDG is an honorary senior clinical lecturer for the university.

## Ethics statement

Ethics approval and informed consent not applicable as this was a literature review.

## Data availability

Not applicable.

## CPD questions

### Learning objective

To gain knowledge on menopause, hormonal status and associated oral disorders.

### Question 1

Which of the following statements about the effect of sex steroids in the mouth is correct?

(a) Oestrogen receptor (ER)‐α is the receptor predominantly seen in the mouth.
(b) ER‐β is the receptor predominantly seen in the mouth.
(c) Oestrogen causes degradation of collagen tissue in the mouth.
(d) Reduction in progesterone levels results in inhibition of gingival fibroblasts.
(e) Sex hormones do not have a direct action on oral tissues.

### Question 2

Which of the following statements about salivary flow in menopause is correct?

(a) Salivary glands do not contain oestrogen receptors and are therefore protected from the fluctuation of hormone levels.
(b) Reduced salivary flow can lead to the development of periodontitis.
(c) Alterations in salivary composition during the menopause include increased levels of sodium in saliva.
(d) Hormone replacement therapy (HRT) has been well established as a treatment for the uncomfortable symptoms of dry mouth.
(e) Salivary flow rates are the same in menstruating and menopausal women.

### Question 3

Which of the following statements about oral lichen planus (OLP) is correct?

(a) OLP is more common in women than men.
(b) OLP affects perimenopausal and premenopausal women equally.
(c) Oestrogen plays a minimal role in T‐cell regulation in immune‐mediated mucocutaneous disease.
(d) Systemic treatment is generally preferred as a first‐line treatment for OLP.
(e) Biologic agents have not been studied as a treatment option for OLP.

### Question 4

According to the literature, which of the following agents has not been reported as a treatment option in oral lichen planus?

(a) Topical tacrolimus 0.1% cream.
(b) Topical triamcinolone acetonide 0.1% gel.
(c) Oral azathioprine 150 mg/day.
(d) Oral gabapentin 300 mg/day.
(e) Photodynamic therapy.

### Question 5

Which of the following statements about burning mouth syndrome (BMS) is true?

(a) Complete resolution of symptoms occurs in 90% of individuals.
(b) Severe cases of BMS do not benefit from anticonvulsants such as gabapentin.
(c) Clonazepam 10 mg tablet held in mouth for 7 min has been shown to reduce symptoms of BMS.
(d) The use of hormone replacement therapy (HRT) in BMS has shown significant reduction in long‐term symptoms.
(e) BMS can occur secondary to drugs, trauma and oral infections.

## Instructions for answering questions

This learning activity is freely available online at http://www.wileyhealthlearning.com/ced

Users are encouraged to

- Read the article in print or online, paying particular attention to the learning points and any author conflict of interest disclosures.
- Reflect on the article.
- Register or login online at http://www.wileyhealthlearning.com/ced and answer the CPD questions.
- Complete the required evaluation component of the activity.

Once the test is passed, you will receive a certificate and the learning activity can be added to your RCP CPD diary as a self‐certified entry.

This activity will be available for CPD credit for 2 years following its publication date. At that time, it will be reviewed and potentially updated and extended for an additional period.

## References

[1] National Institute on Aging . What is menopause? Available at: http://www.nia.nih.gov/health/what‐menopause (accessed 22 November 2021).

[2] Välimaa H , Savolainen S , Soukka T *et al*. Estrogen receptor‐beta is the predominant estrogen receptor subtype in human oral epithelium and salivary glands. J Endocrinol 2004; 180: 55–62. 14709144 10.1677/joe.0.1800055

[3] Scardina GA , Messina P . Oral microcirculation in post‐menopause: a possible correlation with periodontitis. Gerodontology 2012; 29: e1045–51. 22212114 10.1111/j.1741-2358.2011.00608.x

[4] Ciesielska A , Kusiak A , Ossowska A , Grzybowska ME . Changes in the oral cavity in menopausal women—a narrative review. Int J Environ Res Public Health 2022; 19: 253. 10.3390/ijerph19010253 PMC8750983 35010513

[5] Passos‐Soares JS , Vianna MIP , Gomes‐Filho IS *et al*. Association between osteoporosis treatment and severe periodontitis in postmenopausal women. Menopause 2017; 24: 789–95. 28225430 10.1097/GME.0000000000000830

[6] Allen IE , Monroe M , Connelly J *et al*. Effect of postmenopausal hormone replacement therapy on dental outcomes: systematic review of the literature and pharmacoeconomic analysis. Manag Care Interface 2000; 13: 93–9. 11066291

[7] Thompson IOC , van der Bijl P , van Wyk CW , van Eyk AD . A comparative light‐microscopic, electron‐microscopic and chemical study of human vaginal and buccal epithelium. Arch Oral Biol 2001; 46: 1091–8. 11684027 10.1016/s0003-9969(01)00082-6

[8] Emingil G. Menopause and oral microbiome. Report no. [NCT05335733]. Available at: https://clinicaltrials.gov/ct2/show/NCT05335733 (accessed 26 April 2022).

[9] Dodds M , Roland S , Edgar M , Thornhill M . Saliva. A review of its role in maintaining oral health and preventing dental disease. BDJ Team 2015; 2: 11–13.

[10] Mahesh DR , Komali G , Jayanthi K *et al*. Evaluation of salivary flow rate, pH and buffer in pre, post & post menopausal women on HRT. J Clin Diagn Res 2014; 8: 233–6. 10.7860/JCDR/2014/8158.4067 PMC3972571 24701542

[11] Minicucci E , Pires R , Vieira R *et al*. Assessing the impact of menopause on salivary flow and xerostomia. Aust Dent J 2013; 58: 230–4. 23713645 10.1111/adj.12057

[12] Vila T , Rizk AM , Sultan AS , Jabra‐Rizk MA . The power of saliva: antimicrobial and beyond. PLoS Pathog 2019; 15: e1008058. 31725797 10.1371/journal.ppat.1008058 PMC6855406

[13] Shinohara C , Ito K , Takamatsu K *et al*. Factors associated with xerostomia in perimenopausal women. J Obstet Gynaecol Res 2021; 47: 3661–8. 34355462 10.1111/jog.14963

[14] Suri V , Suri V . Menopause and oral health. J Life Health 2014; 5: 115–20. 10.4103/0976-7800.141187 PMC4195183 25316996

[15] Furness S , Worthington HV , Bryan G *et al*. Interventions for the management of dry mouth: topical therapies. Cochrane Database Syst Rev 2011; 12: CD008934. 10.1002/14651858.CD008934.pub2 PMC13266506 22161442

[16] Wang L , Zhu L , Yao Y *et al*. Role of hormone replacement therapy in relieving oral dryness symptoms in postmenopausal women: a case control study. BMC Oral Health 2021; 21: 615. 34861858 10.1186/s12903-021-01966-6 PMC8642912

[17] do Carmo MA , Gleber‐Netto FO , Romano ML *et al*. Clinical and demographic overlaps among immunologically mediated oral diseases: a challenge for clinicians. Gen Dent 2014; 62: 67–72. 24401354

[18] Li C , Tang X , Zheng X *et al*. Global prevalence and incidence estimates of oral lichen planus: a systematic review and meta‐analysis. JAMA Dermatol 2020; 156: 172–81. 31895418 10.1001/jamadermatol.2019.3797 PMC6990670

[19] Bruckmann C . “Oral menopause” – do you know this phenomenon? Womens Health Sci J 2018; 2: 000106.

[20] Oberti L , Alberta L , Massimo P *et al*. Clinical management of oral lichen planus: a systematic review. Mini Rev Med Chem 2019; 19: 1049–59. 30836913 10.2174/1389557519666190301144157

[21] Scardina GA , Messina P , Carini F , Maresi E . A randomized trial assessing the effectiveness of different concentrations of isotretinoin in the management of lichen planus. Int J Oral Maxillofac Surg 2006; 35: 67–71. 16344218 10.1016/j.ijom.2005.05.003

[22] Petruzzi M , Lucchese A , Lajolo C *et al*. Topical retinoids in oral lichen planus treatment: an overview. Dermatology 2013; 226: 61–7. 23548887 10.1159/000346750

[23] Friedlander AH . The physiology, medical management and oral implications of menopause. J Am Dent Assoc 2002; 133: 73–81. 11811747 10.14219/jada.archive.2002.0025

[24] The Menopause Charity . Burning mouth syndrome (BMS). Available at: https://www.themenopausecharity.org/2021/04/24/burning‐mouth‐syndrome‐bms/ (accessed 28 November 2021).

[25] Meurman JH , Tarkkila L , Tiitinen A . The menopause and oral health. Maturitas 2009; 63: 56–62. 19324502 10.1016/j.maturitas.2009.02.009

[26] Bhardwaj A , Bhardwaj SV . Effect of androgens, estrogens and progesterone on periodontal tissues. J Orofac Res 2012; 2: 165–70.

[27] Markou E , Eleana B , Lazaros T , Antonios K . The influence of sex steroid hormones on gingiva of women. Open Dent J 2009; 3: 114–19. 19812718 10.2174/1874210600903010114 PMC2758498

[28] Taneja V . Sex hormones determine immune response. Front Immunol 2018; 9: 1931. 30210492 10.3389/fimmu.2018.01931 PMC6119719

[29] Pfeilschifter J , Köditz R , Pfohl M , Schatz H . Changes in proinflammatory cytokine activity after menopause. Endocr Rev 2002; 23: 90–119. 11844745 10.1210/edrv.23.1.0456

[30] Gupta S , Ghosh S , Gupta S . Interventions for the management of oral lichen planus: a review of the conventional and novel therapies. Oral Dis 2017; 23: 1029–42. 28055124 10.1111/odi.12634

[31] Liu Y , Kim Y , Yoo T *et al*. Burning mouth syndrome: a systematic review of treatments. Oral Dis 2018; 24: 325–34. 28247977 10.1111/odi.12660

[32] White TL , Kent PF , Kurtz DB , Emko P . Effectiveness of gabapentin for treatment of burning mouth syndrome. Arch Otolaryngol Head Neck Surg 2004; 130: 786–8. 15210564 10.1001/archotol.130.6.786

